# Supplementary material for: Association of Coffee and Tea Consumption with the Risk of Asthma: A Prospective Cohort Study from the UK Biobank
Source: Nutrients. 2022 Sep 28;14(19):4039. doi: 10.3390/nu14194039 (PMC9572944; doi:10.3390/nu14194039)
Supplement: Supplementary file 1 [file nutrients-14-04039-s001.zip › nutrients-1939734-supplementary.pdf]

Supplemental Materials

Table S1. Association of coffee and tea with asthma in the UK Biobank cohort by age.

Table S2. Association of coffee and tea with asthma in the UK Biobank cohort by gender.

Table S3. Association of coffee and tea with asthma in the UK Biobank cohort by BMI.

Table S4. Association of coffee and tea with asthma in the UK Biobank cohort by smoking status.

Table S5. Coffee and tea consumption in relation to asthma risk with complete covariates data.

Table S6. Coffee and tea consumption in relation to asthma risk after excluding participants with incident asthma during the first 2 years of follow-up.

Table S1. Association of coffee and tea with asthma in the UK Biobank cohort by age.

Group	Age <60 years				Age ≥ 60 years				*P* value[b]
	Unadjusted HR (95% CI)	*P* value	Multi-adjusted HR (95% CI)[a]	*P* value	Unadjusted HR (95% CI)	*P* value	Multi-adjusted HR (95% CI)[a]	*P* value	
Coffee (cups/d)									
0	1.00 (Ref)		1.00 (Ref)		1.00 (Ref)		1.00 (Ref)		<0.001
0.5-1	0.826(0.760~0.897)	<0.001	0.901(0.829~0.979)	0.014	0.877(0.806~0.955)	0.002	0.948(0.870~1.032)	0.219	
2-3	0.810(0.747~0.877)	<0.001	0.896(0.825~0.973)	0.009	0.789(0.725~0.858)	<0.001	0.870(0.798~0.950)	0.002	
≥4	0.912(0.837~0.995)	0.038	0.963(0.879~1.054)	0.413	0.950(0.866~1.042)	0.274	0.991(0.898~1.093)	0.858	
Tea (cups/d)									
0	1.00 (Ref)		1.00 (Ref)		1.00 (Ref)		1.00 (Ref)		<0.001
0.5-1	0.810(0.722~0.909)	<0.001	0.886(0.789~0.994)	0.040	0.832(0.735~0.942)	0.004	0.895(0.790~1.015)	0.084	
2-3	0.859(0.783~0.941)	0.001	0.945(0.859~1.038)	0.238	0.852(0.774~0.938)	0.001	0.919(0.832~1.016)	0.101	
≥4	0.965(0.887~1.050)	0.413	1.037(0.948~1.135)	0.422	0.919(0.840~1.006)	0.066	0.961(0.872~1.060)	0.427	
Coffee-caffeinated only (cups/day)									
0	1.00 (Ref)		1.00 (Ref)		1.00 (Ref)		1.00 (Ref)		<0.001
0.5-1	0.785(0.718~0.858)	<0.001	0.857(0.783~0.937)	0.001	0.853(0.780~0.934)	0.001	0.918(0.838~1.005)	0.065	
2-3	0.804(0.739~0.875)	<0.001	0.896(0.821~0.977)	0.013	0.758(0.693~0.829)	<0.001	0.832(0.759~0.913)	<0.001	
≥4	0.885(0.807~0.970)	0.009	0.937(0.849~1.033)	0.190	0.907(0.820~1.003)	0.056	0.939(0.844~1.045)	0.247	
Tea or coffee-Caffeine intake quintiles									
Q1(≤160.0)	1.00 (Ref)		1.00 (Ref)		1.00 (Ref)		1.00 (Ref)		<0.001
Q2(160.0-235.0)	0.849(0.774~0.932)	0.001	0.866(0.789~0.951)	0.003	0.888(0.808~0.976)	0.014	0.910(0.827~1.000)	0.051	

Q3(235.0-305.0)	0.891(0.812~0.977)	0.014	0.886(0.806~0.974)	0.012	0.915(0.832~1.006)	0.066	0.919(0.834~1.013)	0.089
Q4(305.0-390.0)	0.900(0.822~0.986)	0.023	0.884(0.804~0.972)	0.011	0.934(0.852~1.025)	0.150	0.931(0.846~1.025)	0.145
Q5 (≥390.0)	1.034(0.948~1.129)	0.447	0.935(0.849~1.030)	0.174	1.011(0.920~1.112)	0.815	0.952(0.859~1.056)	0.354

[a]Multivariable model is adjusted for sex, race, qualification, smoking status, Townsend deprivation, body mass index, and we adjusted for coffee in tea analysis or for tea in coffee analysis. [b]*P* value for heterogeneity corresponds to the χ^2 test statistic for the likelihood ratio test comparing the models with and without the interaction term between coffee, tea, or their combination (categorical) and the stratifying variable of interest (categorical). Abbreviations: HR: Hazard Ratio; CI, Confidence Interval; Q, quartiles.

Table S2. Association of coffee and tea with asthma in the UK Biobank cohort by gender.

Group	Male				Female				*P* value[b]
	Unadjusted HR (95% CI)	*P* value	Multi-adjusted HR (95% CI)[a]	*P* value	Unadjusted HR (95% CI)	*P* value	Multi-adjusted HR (95% CI)[a]	*P* value	
Coffee (cups/d)									
0	1.000 (Ref)		1.000 (Ref)		1.000 (Ref)		1.000 (Ref)		<0.001
0.5-1	0.845(0.768~0.929)	0.001	0.891(0.810~0.981)	0.019	0.892(0.828~0.962)	0.003	0.942(0.874~1.017)	0.125	
2-3	0.800(0.730~0.877)	<0.001	0.868(0.790~0.955)	0.004	0.840(0.780~0.906)	<0.001	0.888(0.822~0.959)	0.003	
≥4	0.949(0.862~1.045)	0.285	1.013(0.916~1.121)	0.799	0.960(0.883~1.045)	0.345	0.944(0.863~1.032)	0.206	
Tea (cups/d)									
0	1.000 (Ref)		1.000 (Ref)		1.000 (Ref)		1.000 (Ref)		<0.001
0.5-1	0.888(0.781~1.011)	0.073	0.941(0.826~1.072)	0.363	0.785(0.702~0.878)	<0.001	0.851(0.761~0.953)	0.005	
2-3	0.900(0.810~0.999)	0.049	0.933(0.836~1.040)	0.211	0.857(0.787~0.933)	<0.001	0.929(0.850~1.015)	0.102	
≥4	0.968(0.878~1.068)	0.520	0.964(0.868~1.071)	0.493	0.962(0.889~1.040)	0.329	1.013(0.931~1.103)	0.764	
Coffee-caffeinated only (cups/day)									
0	1.00 (Ref)		1.00 (Ref)		1.00 (Ref)		1.00 (Ref)		<0.001
0.5-1	0.842(0.763~0.930)	0.001	0.889(0.804~0.983)	0.021	0.842(0.776~0.913)	<0.001	0.885(0.815~0.961)	0.004	
2-3	0.790(0.718~0.870)	<0.001	0.862(0.781~0.952)	0.003	0.817(0.754~0.885)	<0.001	0.862(0.793~0.936)	<0.001	
≥4	0.939(0.849~1.038)	0.218	1.006(0.904~1.118)	0.918	0.901(0.821~0.989)	0.029	0.879(0.796~0.971)	0.011	
Tea or coffee-Caffeine intake quintiles									

Q1(≤160.0)	1.00 (Ref)		1.00 (Ref)		1.00 (Ref)		1.00 (Ref)		<0.001
Q2(160.0-235.0)	0.975(0.877~1.085)	0.646	0.982(0.883~1.093)	0.742	0.845(0.777~0.919)	<0.001	0.855(0.785~0.930)	<0.001	
Q3(235.0-305.0)	0.993(0.893~1.105)	0.902	1.002(0.900~1.115)	0.975	0.889(0.818~0.967)	0.006	0.886(0.814~0.964)	0.005	
Q4(305.0-390.0)	1.020(0.920~1.130)	0.709	1.026(0.924~1.138)	0.635	0.907(0.835~0.985)	0.021	0.894(0.822~0.972)	0.009	
Q5 (≥390.0)	1.078(0.975~1.192)	0.143	1.051(0.949~1.164)	0.340	1.056(0.971~1.148)	0.207	0.981(0.901~1.069)	0.664	

[a] Multivariable model is adjusted for age, race, qualification, smoking status, Townsend deprivation, body mass index, and we adjusted for coffee in tea analysis or for tea in coffee analysis. [b] *P* value for heterogeneity corresponds to the χ^2 test statistic for the likelihood ratio test comparing the models with and without the interaction term between coffee, tea, or their combination (categorical) and the stratifying variable of interest (categorical). Abbreviations: HR: Hazard Ratio; CI, Confidence Interval; Q, quartiles.

Table S3. Association of coffee and tea with asthma in the UK Biobank cohort by BMI.

Group	BMI <25 kg/m^2				BMI 25 to <30 kg/m^2				BMI ≥30 kg/m^2				
	**Unadjusted HR (95% CI)**	***P* value**	**Multi-adjusted HR (95% CI)[a]**	***P* value**	**Unadjusted HR (95% CI)**	***P* value**	**Multi-adjusted HR (95% CI)[a]**	***P* value**	**Unadjusted HR (95% CI)**	***P* value**	**Multi-adjusted HR (95% CI)[a]**	***P* value**	***P* value[b]**
Coffee (cups/d)													
0	1.00 (Ref)		1.00 (Ref)		1.00 (Ref)		1.00 (Ref)		1.00 (Ref)		1.00 (Ref)		<0.001
0.5-1	0.883(0.788~0.990)	0.032	0.909(0.810~1.020)	0.105	0.894(0.814~0.982)	0.019	0.914(0.832~1.005)	0.063	0.900(0.813~0.996)	0.043	0.936(0.845~1.037)	0.207	
2-3	0.832(0.742~0.933)	0.002	0.865(0.768~0.973)	0.015	0.838(0.764~0.918)	<0.001	0.885(0.805~0.973)	0.012	0.808(0.732~0.893)	<0.001	0.872(0.787~0.966)	0.009	
≥4	0.883(0.772~1.010)	0.070	0.866(0.752~0.997)	0.046	0.942(0.853~1.040)	0.237	1.013(1.008~1.018)	0.809	0.902(0.813~1.000)	0.050	0.991(0.889~1.106)	0.878	
Tea (cups/d)													
0	1.00 (Ref)		1.00 (Ref)		1.00 (Ref)		1.00 (Ref)		1.00 (Ref)		1.00 (Ref)		<0.001
0.5-1	0.841(0.709~0.998)	0.048	0.879(0.740~1.044)	0.141	0.765(0.667~0.877)	<0.001	0.790(0.688~0.907)	0.001	0.949(0.827~1.090)	0.458	0.994(0.865~1.142)	0.930	
2-3	0.838(0.730~0.961)	0.012	0.853(0.740~0.983)	0.028	0.893(0.804~0.991)	0.033	0.900(0.807~1.003)	0.056	0.979(0.877~1.093)	0.709	0.991(0.884~1.110)	0.876	
≥4	0.997(0.878~1.133)	0.965	0.971(0.848~1.113)	0.673	0.950(0.861~1.047)	0.298	0.936(0.843~1.040)	0.219	1.042(0.942~1.153)	0.420	1.031(0.925~1.150)	0.577	
Coffee-caffeinated only (cups/day)													
0	1.00 (Ref)		1.00 (Ref)		1.00 (Ref)		1.00 (Ref)		1.00 (Ref)		1.00 (Ref)		<0.00

													1
0.5-1	0.855(0.757~0.965)	0.011	0.878(0.777~0.993)	0.039	0.858(0.777~0.948)	0.003	0.879(0.795~0.972)	0.012	0.854(0.766~0.953)	0.005	0.898(0.805~1.003)	0.055	
2-3	0.805(0.713~0.909)	<0.001	0.838(0.739~0.950)	0.006	0.828(0.752~0.912)	<0.001	0.876(0.793~0.968)	0.010	0.778(0.700~0.866)	<0.001	0.847(0.759~0.946)	0.003	
≥4	0.837(0.723~0.970)	0.018	0.814(0.698~0.950)	0.009	0.931(0.837~1.035)	0.183	0.998(0.892~1.117)	0.971	0.845(0.755~0.945)	0.003	0.936(0.831~1.055)	0.281	
Tea or coffee-Caffeine intake quintiles													
Q1(≤160.0)	1.00 (Ref)		1.00 (Ref)		1.00 (Ref)		1.00 (Ref)		1.00 (Ref)		1.00 (Ref)		<0.001
Q2(160.0-235.0)	0.872(0.768~0.990)	0.034	0.857(0.755~0.974)	0.018	0.946(0.852~1.050)	0.297	0.943(0.849~1.048)	0.278	0.865(0.772~0.970)	0.013	0.878(0.783~0.985)	0.026	
Q3(235.0-305.0)	0.903(0.794~1.028)	0.123	0.880(0.773~1.002)	0.053	0.961(0.866~1.067)	0.461	0.961(0.865~1.068)	0.457	0.903(0.807~1.011)	0.078	0.912(0.813~1.022)	0.113	
Q4(305.0-390.0)	0.929(0.818~1.055)	0.254	0.896(0.788~1.018)	0.092	0.968(0.874~1.072)	0.536	0.968(0.873~1.073)	0.533	0.911(0.816~1.017)	0.097	0.930(0.833~1.040)	0.203	
Q5 (≥390.0)	1.024(0.898~1.167)	0.726	0.924(0.808~1.056)	0.244	1.025(0.925~1.136)	0.639	1.028(0.926~1.140)	0.610	0.985(0.886~1.095)	0.778	1.013(0.909~1.128)	0.818	

[a]Multivariable model is adjusted for age, sex, race, qualification, smoking status, Townsend deprivation, and we adjusted for coffee in tea analysis or for tea in coffee analysis. [b]*P* value for heterogeneity corresponds to the χ^2 test statistic for the likelihood ratio test comparing the models with and without the interaction term between coffee, tea, or their combination (categorical) and the stratifying variable of interest (categorical). Abbreviations: BMI, body mass index; HR: Hazard Ratio; CI, Confidence Interval; Q, quartiles.

Table S4. Association of coffee and tea with asthma in the UK Biobank cohort by smoking status.

Group	Smoking status-never				Smoking status-former				Smoking status-current				
	**Unadjusted HR (95% CI)**	***P* value**	**Multi-adjusted HR (95% CI)[a]**	***P* value**	**Unadjusted HR (95% CI)**	***P* value**	**Multi-adjusted HR (95% CI)[a]**	***P* value**	**Unadjusted HR (95% CI)**	***P* value**	**Multi-adjusted HR (95% CI)[a]**	***P* value**	***P* value[b]**
Coffee (cups/d)													
0	1.00 (Ref)		1.00 (Ref)		1.00 (Ref)		1.00 (Ref)		1.00 (Ref)		1.00 (Ref)		
0.5-1	0.899(0.829~0.975)	0.010	0.943(0.868~1.023)	0.157	0.854(0.775~0.942)	0.002	0.897(0.812~0.990)	0.030	0.812(0.678~0.972)	0.023	0.910(0.759~1.093)	0.314	<0.001
2-3	0.827(0.762~0.897)	<0.001	0.881(0.811~0.958)	0.003	0.816(0.742~0.897)	<0.001	0.871(0.789~0.961)	0.006	0.722(0.610~0.855)	<0.001	0.862(0.723~1.026)	0.095	
≥4	0.983(0.896~1.078)	0.716	1.022(0.928~1.126)	0.655	0.847(0.763~0.941)	0.002	0.882(0.789~0.986)	0.028	0.841(0.719~0.985)	0.031	1.022(0.864~1.208)	0.801	
Tea (cups/d)													
0	1.00 (Ref)		1.00 (Ref)		1.00 (Ref)		1.00 (Ref)		1.00 (Ref)		1.00 (Ref)		<0.001
0.5-1	0.817(0.723~0.923)	0.001	0.869(0.769~0.982)	0.024	0.905(0.789~1.037)	0.149	0.977(0.851~1.120)	0.737	0.668(0.528~0.845)	0.001	0.753(0.594~0.955)	0.019	
2-3	0.902(0.821~0.992)	0.033	0.948(0.860~1.045)	0.285	0.906(0.789~1.037)	0.078	0.943(0.841~1.056)	0.310	0.729(0.606~0.878)	0.001	0.809(0.667~0.981)	0.032	
≥4	0.956(0.874~1.045)	0.319	0.988(0.899~1.086)	0.806	0.968(0.874~1.071)	0.526	0.964(0.863~1.077)	0.513	1.025(0.880~1.193)	0.753	1.084(0.916~1.284)	0.349	
Coffee-caffeinated only (cups/day)													
0	1.00 (Ref)		1.00 (Ref)		1.00 (Ref)		1.00 (Ref)		1.00 (Ref)		1.00 (Ref)		<0.001

0.5-1	0.837(0.767~0.915)	<0.001	0.880(0.805~0.962)	0.005	0.852(0.769~0.944)	0.002	0.897(0.809~0.995)	0.040	0.760(0.627~0.920)	0.005	0.858(0.707~1.041)	0.121	
2-3	0.810(0.743~0.884)	<0.001	0.869(0.795~0.950)	0.002	0.783(0.708~0.865)	<0.001	0.840(0.757~0.932)	0.001	0.703(0.590~0.839)	<0.001	0.849(0.707~1.019)	0.078	
≥4	0.957(0.865~1.059)	0.393	1.000(0.900~1.111)	0.998	0.790(0.705~0.886)	<0.001	0.828(0.733~0.934)	0.002	0.806(0.685~0.949)	0.010	0.986(0.828~1.173)	0.872	
Tea or coffee-Caffeine intake quintiles													
Q1(≤160.0)	1.00 (Ref)		1.00 (Ref)		1.00 (Ref)		1.00 (Ref)		1.00 (Ref)		1.00 (Ref)		<0.001
Q2(160.0-235.0)	0.848(0.775~0.927)	<0.001	0.867(0.792~0.949)	0.002	0.914(0.822~1.017)	0.099	0.926(0.832~1.031)	0.162	0.951(0.752~1.202)	0.672	0.995(0.787~1.259)	0.968	
Q3(235.0-305.0)	0.856(0.781~0.938)	0.001	0.872(0.796~0.957)	0.004	0.939(0.844~1.046)	0.253	0.945(0.848~1.052)	0.302	1.147(0.923~1.425)	0.217	1.191(0.957~1.482)	0.118	
Q4(305.0-390.0)	0.915(0.837~1.000)	0.049	0.932(0.852~1.020)	0.124	0.870(0.781~0.968)	0.011	0.880(0.790~0.981)	0.021	1.229(1.002~1.507)	0.048	1.264(1.029~1.552)	0.026	
Q5 (≥390.0)	0.998(0.909~1.095)	0.960	1.004(0.913~1.103)	0.938	0.935(0.840~1.041)	0.222	0.934(0.838~1.040)	0.214	1.224(1.015~1.477)	0.035	1.243(1.029~1.502)	0.024	

[a]Multivariable model is adjusted for age, sex, race, qualification, Townsend deprivation, body mass index, and we adjusted for coffee in tea analysis or for tea in coffee analysis. [b]*P* value for heterogeneity corresponds to the χ^2 test statistic for the likelihood ratio test comparing the models with and without the interaction term between coffee, tea, or their combination (categorical) and the stratifying variable of interest (categorical). Abbreviations: HR: Hazard Ratio; CI, Confidence Interval; Q, quartiles.

Table S5. Coffee and tea consumption in relation to asthma risk with complete covariates data.

	Hazard ratio for Asthma							
Characteristics	HR (95%CI) from Crude Model[a]	*p*-Value	HR (95%CI) from Model 1[b]	*p*-Value	HR (95%CI) from Model 2[c]	*p*-Value	HR (95%CI) from Model 3[d]	*p*-Value
**Coffee (cups/day)**								
0	Ref		Ref		Ref		Ref	
0.5 to 1	0.870(0.820~0.923)	<0.001	0.847 (0.798~0.899)	<0.001	0.910 (0.857~0.965)	0.002	0.918 (0.865~0.974)	0.005
2 to3	0.817 (0.771~0.866)	<0.001	0.800 (0.754~0.847)	<0.001	0.855 (0.807~0.907)	<0.001	0.877 (0.826~0.931)	<0.001
≥4	0.938 (0.881~0.999)	0.047	0.942 (0.884~1.003)	0.063	0.930 (0.873~0.992)	0.026	0.968 (0.906~1.035)	0.345
	Ptrend=0.002		Ptrend=0.003		Ptrend=0.002		Ptrend=0.083	
**Tea (cups/day)**								
0	Ref		Ref		Ref		Ref	
0.5 to 1	0.822 (0.755~0.894)	<0.001	0.827 (0.760~0.900)	<0.001	0.891 (0.819~0.970)	0.008	0.889 (0.816~0.967)	0.006
2 to3	0.871 (0.815~0.931)	<0.001	0.857 (0.802~0.915)	<0.001	0.936 (0.876~1.000)	0.052	0.930 (0.868~0.996)	0.038
≥4	0.961 (0.903~1.021)	0.200	0.938 (0.882~0.998)	0.043	1.000 (0.941~1.064)	0.988	0.991 (0.928~1.059)	0.797
	Ptrend=0.579		Ptrend=0.710				Ptrend=0.418	
**Coffee-caffeinated only (cups/day)**								
0	Ref		Ref		Ref		Ref	
0.5 to 1	0.835 (0.784~0.889)	<0.001	0.817 (0.767~0.871)	<0.001	0.874 (0.820~0.931)	<0.001	0.881 (0.827~0.939)	<0.001
2 to3	0.796 (0.749~0.847)	<0.001	0.784 (0.737~0.833)	<0.001	0.837 (0.786~0.890)	<0.001	0.858 (0.805~0.915)	<0.001
≥4	0.900 (0.841~0.963)	0.002	0.909 (0.849~0.973)	0.006	0.894 (0.835~0.958)	0.002	0.931 (0.866~1.001)	0.052
	Ptrend<0.001		Ptrend<0.001		Ptrend<0.001		Ptrend=0.006	
**Tea and coffee-Caffeine intake quintiles**								
Q1(≤160.0)	Ref		Ref		Ref			
Q2(160.0-235.0)	0.890 (0.833~0.951)	0.001	0.867 (0.812~0.926)	<0.001	0.901 (0.843~0.963)	0.002	NA	
Q3(235.0-305.0)	0.922 (0.863~0.985)	0.015	0.897 (0.840~0.959)	0.001	0.926 (0.866~0.989)	0.023	NA	

Q4 (305.0-390.0)	0.939 (0.881~1.002)	0.057	0.914 (0.857~0.976)	0.007	0.939 (0.880~1.002)	0.059	NA
Q5 (≥390.0）	1.034 (0.969~1.102)	0.311	1.035 (0.971~1.104)	0.291	0.998 (0.935~1.065)	0.951	NA
	P-trend=0.180		Ptrend=0.190		Ptrend=0.765		

[a] unadjusted (crude) model; [b] Adjusted by age and gender; [c] Adjusted by age, gender, race, body mass index, smoking status, education, and Townsend Index; [d] Adjusted by age, gender, race, body mass index, smoking status, education, and Townsend Index, and adjusted for coffee in tea analysis or for tea in coffee analysis. Abbreviations: HR: Hazard Ratio; CI, Confidence Interval; Q, quartiles.

Table S6. Coffee and tea consumption in relation to asthma risk after excluding participants with incident asthma during the first 2 years of follow-up.

	Hazard ratio for Asthma							
Characteristics	HR (95%CI) from Crude Model[a]	*p*-Value	HR (95%CI) from Model 1[b]	*p*-Value	HR (95%CI) from Model 2[c]	*p*-Value	HR (95%CI) from Model 3[d]	*p*-Value
**Coffee (cups/day)**								
0	Ref		Ref		Ref		Ref	
0.5 to 1	0.859 (0.804~0.919)	<0.001	0.836 (0.782~0.894)	<0.001	0.895 (0.837~0.957)	0.001	0.903 (0.844~0.965)	0.003
2 to3	0.817 (0.765~0.872)	<0.001	0.799 (0.748~0.853)	<0.001	0.851 (0.796~0.908)	<0.001	0.871 (0.815~0.932)	<0.001
≥4	0.928 (0.864~0.997)	0.040	0.932 (0.868~1.001)	0.055	0.916 (0.852~0.984)	0.017	0.952 (0.883~1.026)	0.197
	Ptrend=0.004		Ptrend=0.005		Ptrend=0.002		Ptrend=0.061	
**Tea (cups/day)**								
0	Ref		Ref		Ref		Ref	
0.5 to 1	0.816 (0.742~0.898)	<0.001	0.822 (0.747~0.905)	<0.001	0.886 (0.805~0.975)	0.013	0.882 (0.801~0.971)	0.011
2 to3	0.886 (0.822~0.954)	0.001	0.870 (0.808~0.938)	<0.001	0.951 (0.883~1.026)	0.195	0.942 (0.872~1.018)	0.133
≥4	0.964 (0.899~1.033)	0.295	0.941 (0.878~1.009)	0.085	1.003 (0.936~1.076)	0.923	0.990 (0.918~1.067)	0.788
	Ptrend=0.526		Ptrend=0.836		Ptrend=0.245		Ptrend=0.480	
**Coffee-caffeinated only (cups/day)**								
0	Ref		Ref		Ref		Ref	
0.5 to 1	0.826 (0.769~0.887)	<0.001	0.809 (0.753~0.869)	<0.001	0.862 (0.803~0.926)	<0.001	0.869 (0.809~0.934)	<0.001
2 to3	0.789 (0.736~0.845)	<0.001	0.777 (0.725~0.833)	<0.001	0.826 (0.770~0.885)	<0.001	0.847 (0.788~0.910)	<0.001
≥4	0.900 (0.834~0.972)	0.007	0.911 (0.844~0.984)	0.018	0.890 ((0.824~0.962)	0.003	0.926 (0.854~1.005)	0.065
	Ptrend<0.001		Ptrend<0.001		Ptrend<0.001		Ptrend=0.008	
**Tea and coffee-Caffeine intake quintiles**								
Q1(≤160.0)	Ref		Ref		Ref			
Q2(160.0-235.0)	0.906 (0.841~0.976)	<0.001	0.882 (0.819~0.950)	0.001	0.914 (0.848~0.985)	0.018	NA	

Q3(235.0-305.0)	0.914 (0.848~0.985)	0.018	0.889 (0.825~0.958)	0.002	0.913 (0.847~0.985)	0.019	NA
Q4 (305.0-390.0)	0.962 (0.895~1.035)	0.300	0.936 (0.871~1.007)	0.077	0.957 (0.890~1.030)	0.242	NA
Q5 (≥390.0）	1.039 (0.966~1.117)	0.304	1.041 (0.968~1.120)	0.276	0.999 (0.928~1.076)	0.979	NA
	P-trend=0.158		Ptrend=0.160		Ptrend=0.712		

[a] unadjusted (crude) model; [b] Adjusted by age and gender; [c] Adjusted by age, gender, race, body mass index, smoking status, education, and Townsend Index; [d] Adjusted by age, gender, race, body mass index, smoking status, education, and Townsend Index, and adjusted for coffee in tea analysis or for tea in coffee analysis. Abbreviations: HR: Hazard Ratio; CI, Confidence Interval; Q, quartiles.
